# Supplementary material for: Upcycling Compact Discs for Flexible and Stretchable Bioelectronic Applications
Source: Nat Commun. 2022 Jun 28;13:3727. doi: 10.1038/s41467-022-31338-9 (PMC9240022; doi:10.1038/s41467-022-31338-9)
Supplement: Supplementary file 1 — Supplementary Information [file 41467_2022_31338_MOESM1_ESM.pdf]

Supplementary Information

Upcycling Compact Discs for Flexible and Stretchable Bioelectronic Applications

Matthew S. Brown[1], Louis Somma[1], Melissa Mendoza[1], Yeonsik Noh[2], Gretchen J. Mahler[1], and Ahyeon Koh[1*]

[1]Department of Biomedical Engineering, State University of New York at Binghamton, Binghamton, NY 13902, USA
[2]College of Nursing and Department of Electrical and Computer Engineering, University of Massachusetts, Amherst, MA 01003, USA

[*]Corresponding author. Email: akoh@binghamton.edu

Supplementary Note 1: Alternative Fabrication

To decrease the oval thickness of the metal layer, the processing of the CD can be altered by the removal of the protective layer. The protective layer can be removed by soaking in hydrochloric acid (HCl) or nitric acid (HNO_3). After 10 minutes in non-diluted HCl, or 3.5 min in 3:1 HNO_3:DI water, the protective layer of the CD is removed. The HCl soak produced a thickness of ~70.04 ± 3.51 nm and the HNO_3 soak, a thickness of ~18.96 ± 5.28 nm (Supplementary Fig. 8e-f). Scanning electron microscope (SEM) images of the removed protective layer reveal the buckles in the metal layer that were engineered into the CDs for data storage, called "lands" and "pits". The FTIR spectrum of the protective layer after being soaked in HCl and HNO_3 confirms the full removal of the layer, where the characteristic peaks are no longer present, and a metal-carbonyl complex can be seen (Supplementary Fig. 6c). The energy-dispersive X-ray spectroscopy (EDS) of the metal layer after the acetone, HCl, and HNO_3 soak was analyzed (Supplementary Fig. 10). After the acetone and HCl soak, Ag and Au can be seen within the spectrum at similar concentrations 70.95 and 29.05 wt.% (Supplementary Fig. 10a-d). However, since Ag is dissolved in HNO_3, only Au can be seen by EDS analysis (Supplementary Fig. 10e-f). The ultra-thin metal layer creates difficulties during processing while trying to maintain the structural integrity of the film.

Supplementary Note 2: Calibration of Stretchable Resistive Temperature Detector Sensor

Body temperature is a vital sign commonly used in clinical settings to access the current state of a patient's health. Here, we created upcycled CD electronic (UCDE) temperature sensors that can be used to measure on-skin, real-time temperature. The stretchable resistive temperature detector (RTD) sensor was calibrated by placing it on a hotplate next to a thermocouple and recording the

associated 4 probe resistivity with the temperature reading from the thermocouple. The relationship between the resistivity changes linearly proportional to the temperature that can be denoted by the following equation:

$$R = R_0(1 + \alpha(T - T_0)) \quad (1)$$

where R is the changed resistances, R_0 is the initial resistance, α is the temperature coefficient of resistance (TCR), T is the measured temperature, and T_0 is the initial temperature[1]. The results are shown in Fig. 3d. The TCR of the UCDE RTD was measured to be 0.0009 °C^{-1} at 20 °C whereas the TCR of gold is 0.0037 °C^{-1} at 20 °C. We believe there is a large variation from the TCR of gold because the CD metal isn't a pure gold metal film and due to the ultrathin metal film, surface scattering may occur.

The performance of the calibrated UCDE RTD sensor was compared to the FLIR camera in real-time. Four probe resistance of the UCDE RTD was continuously acquired by a digital multimeter (Keysight, 34460A). Results are presented in Fig. 3e. The maximum error of temperature measurement using the UCDE RTD is ± 6.75 °C seen at the hyperpolarization of the temperature curve that could be due to the UCDE recording the temperature more accurately as the heat dissipates through the ambient air.

Supplementary Note 3: Fabrication of Biodegradable, Transient Electronics

The biodegradable electronics were upcycled through a process illustrated in Supplementary Fig. 19. This process required the full removal of the plastic layer on the CD (Supplementary Fig. 5). To remove the plastic layer, the CD was soaked in nitric acid and the plastic layer was delaminated from the metal layer of the CD (Supplementary Fig. 19a and 20). The exposed metal layer from the CD (~18.96 ± 5.28 nm thick) was composed of Au as confirmed by SEM EDX

(Supplementary Fig. 8f and Fig. 10f). This nitric acid soak removed the Ag present in the metal layer leaving behind a film of Au. A solution of 5% PVA was casted and coated with a 50 µm thick, thin-film bar coater and curing at 80 °C for 1 hr. The PCL-based device was fabricated by reflowing PCL above its melting temperature at 57 °C while coating with a 50 µm thick, thin-film bar coater and allowing for cooling and recrystallization (Supplementary Fig. 19b). Advanced patterning and structures can be developed with this layout, as PCL has been demonstrated to have strong adhesion to gold and utilized with advanced microfabrication techniques[2]. Next, they were detached from the CD leaving behind a metal-PVA and/or metal-PCL device (Supplementary Fig. 19c). The resistor shape was then patterned into the device by etching with a mechanical cutter and removing the excess (Supplementary Fig. 19d-e).

Supplementary Discussion: Solutions to Recycle Acetone and Polycarbonate

The UCDEs were upcycled with acetone to break down the polycarbonate and allow for the simple release of the metal layer. The minimum volume required is 40 mL and can be reused with multiple CDs. The procedure requires the CDs soaked for a short period of time, thus, the polycarbonate dissolved within acetone is less than µg concentrations and even soaking the CD for 1 hour, no large peak changes can be detected by FTIR (Supplementary Fig. 6b). After the metal layer is stripped from the CD, the polycarbonate substrate remains. The CD can no longer be classified as laced e-waste (mixed plastic and metal) and can be recycled through conventional methods. Here we discuss solutions to recycle the acetone and polycarbonate substrate.

For decades acetone has been used by many industries to clean large scale and laboratory equipment. When acetone is used and contaminated it is shipped to off-site locations for proper disposal. Recovery and recycling of acetone can be utilized to alleviate the cost and environmental

impact of disposing acetone. Through simple distillation, this process can be operated through a continuous or batchwise process[3]. Solvent recyclers (e.g., NexGen Enviro Systems, Inc.) are commercially available, allowing for high-performance removal of contaminants and recovery of the solvent. However, these systems are quite expensive. To alleviate these costs, Zweckmair et al. details a simple automated process to recycle acetone through a home-built distillation unit[4].

Many plastic recycling companies process polycarbonate. The polycarbonate is processed accordingly: (1) shredded and grinded into granules, (2) washed to clean contaminates, (3) densified, (4) blended and reprocessed into a resin or pellet, (5) compounded with additives. Researchers have also been looking for solutions to address this issue at the lab scale. Jones et al. recently developed a method to synthesis poly(aryl ether sulfone)s (PSUs) from the depolymerization of polycarbonate CDs[5]. The high performance engineered thermoplastics, PSUs can be repurposed for medical equipment or water purification applications[5].

Supplementary Methods: Electrochemical Performance Evaluation

Chronoamperometry was used to evaluate the sensitivity, dynamic range, and response time of the UCDE amperometric sensors. The electrochemical analysis was conducted with the UCDE reference, counter, and working electrode at room temperature in 1x PBS (pH 7.4). The amperometric response for the enzymatic sensors was monitored by injecting set concentrations of L-lactate or glucose. The oxygen sensor was monitored and modulated by changing the gas concentration in solution by changing the ratio of oxygen and nitrogen gas with a proportioner and monitoring the dissolved oxygen with a DO meter (Hanna Instruments). The applied potentials were 0.0 V vs. fabricated Ag/AgCl for lactate and glucose sensing and -0.4 V vs. fabricated Ag/AgCl for oxygen sensing.

Open circuit potential (OCP) was used to determine the performance of the pH sensor. The electrochemical cell was the UCDE reference, counter, and working electrode. The (OCP) response was monitored by injecting pH buffer solutions in an aqueous bath while monitoring the pH changes with a pH meter (Hanna Instruments).

Supplementary Figures

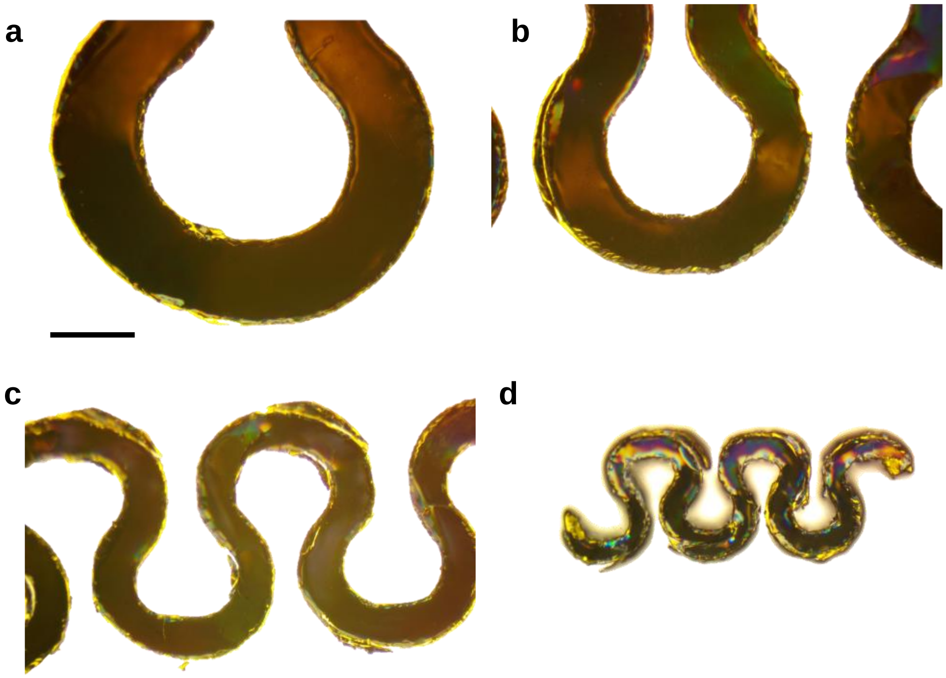

Supplementary Fig. 1. Patterning wire resolution. Various feature sizes: (**a**) 1 mm (scale bar, 1 mm), (**b**) 0.75 mm, (**c**) 0.5 mm, and (**d**) 0.25 mm.

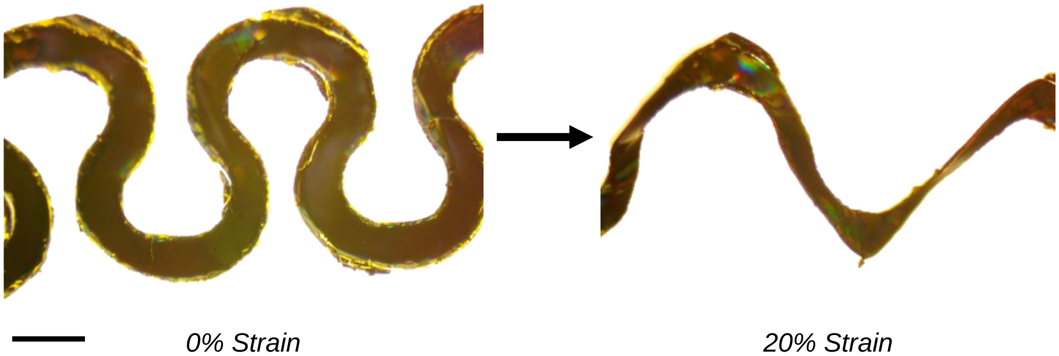

Supplementary Fig. 2. Photograph of the UCDEs during stretching (scale bar 1 mm).

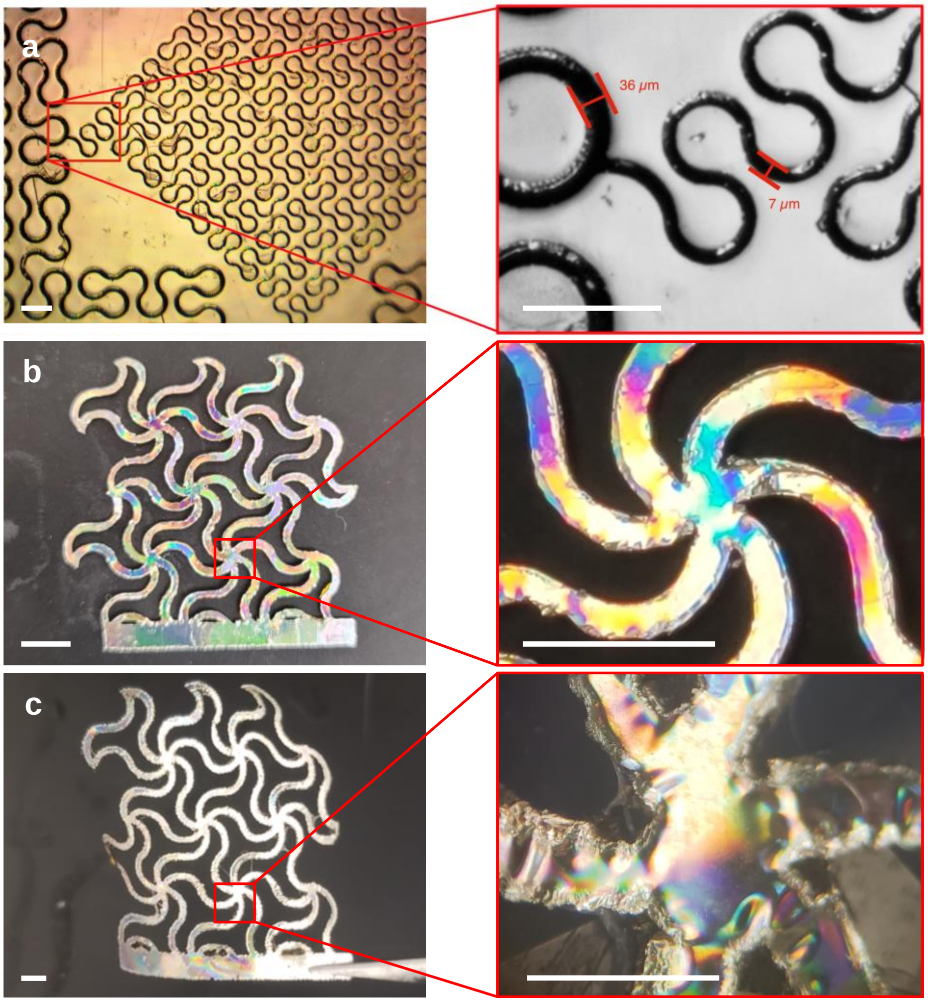

Supplementary Fig. 3. Various patterning methods for the UCDEs. (**a**) Photolithography (scale bar, 200 μm). (**b**) Mechanical cutter (scale bar, 4 mm) (**c**) CO_2 laser engraver (scale bar, 2 mm).

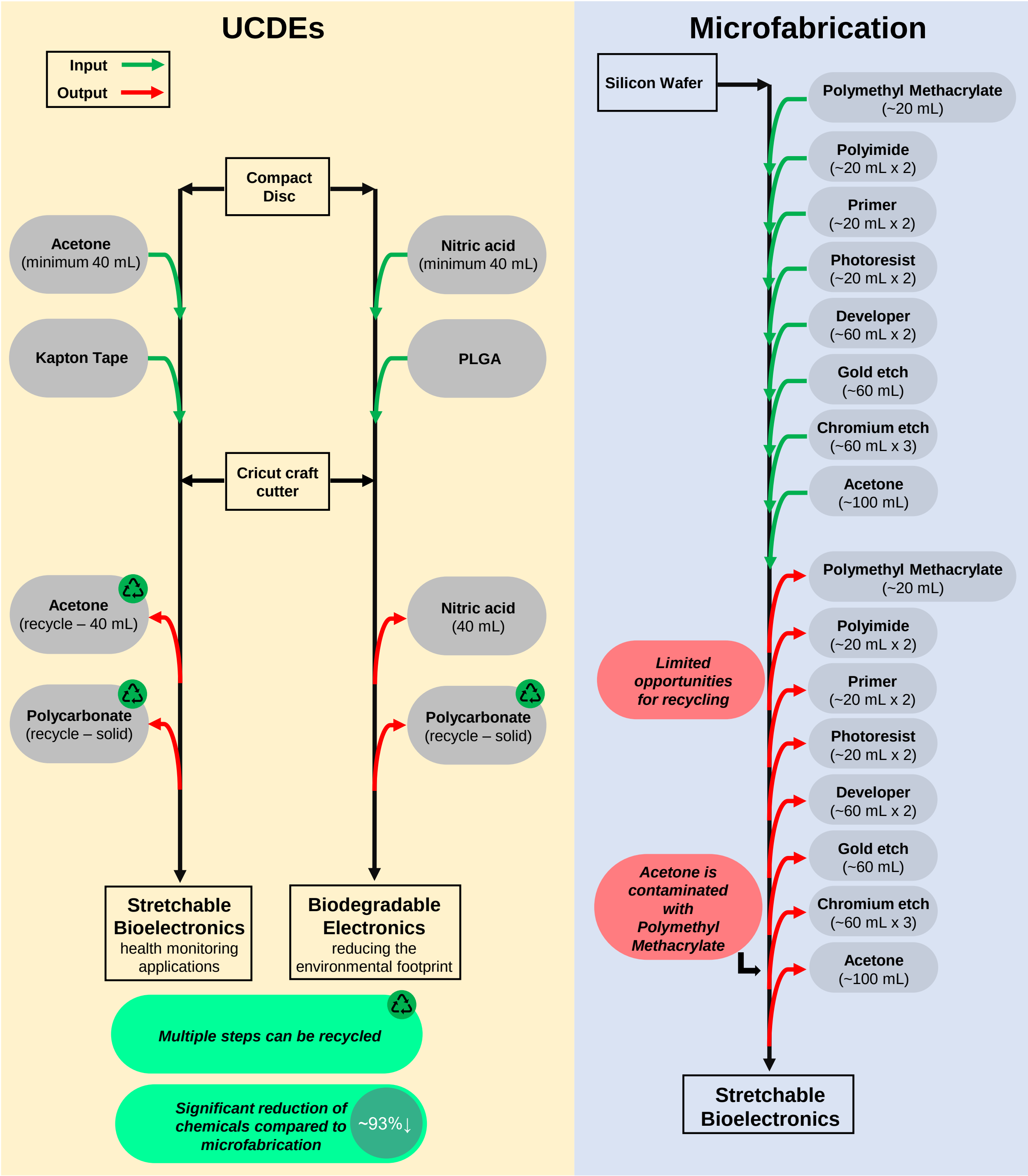

Supplementary Fig. 4. Inputs and outputs of the upcycling process vs. microfabrication.

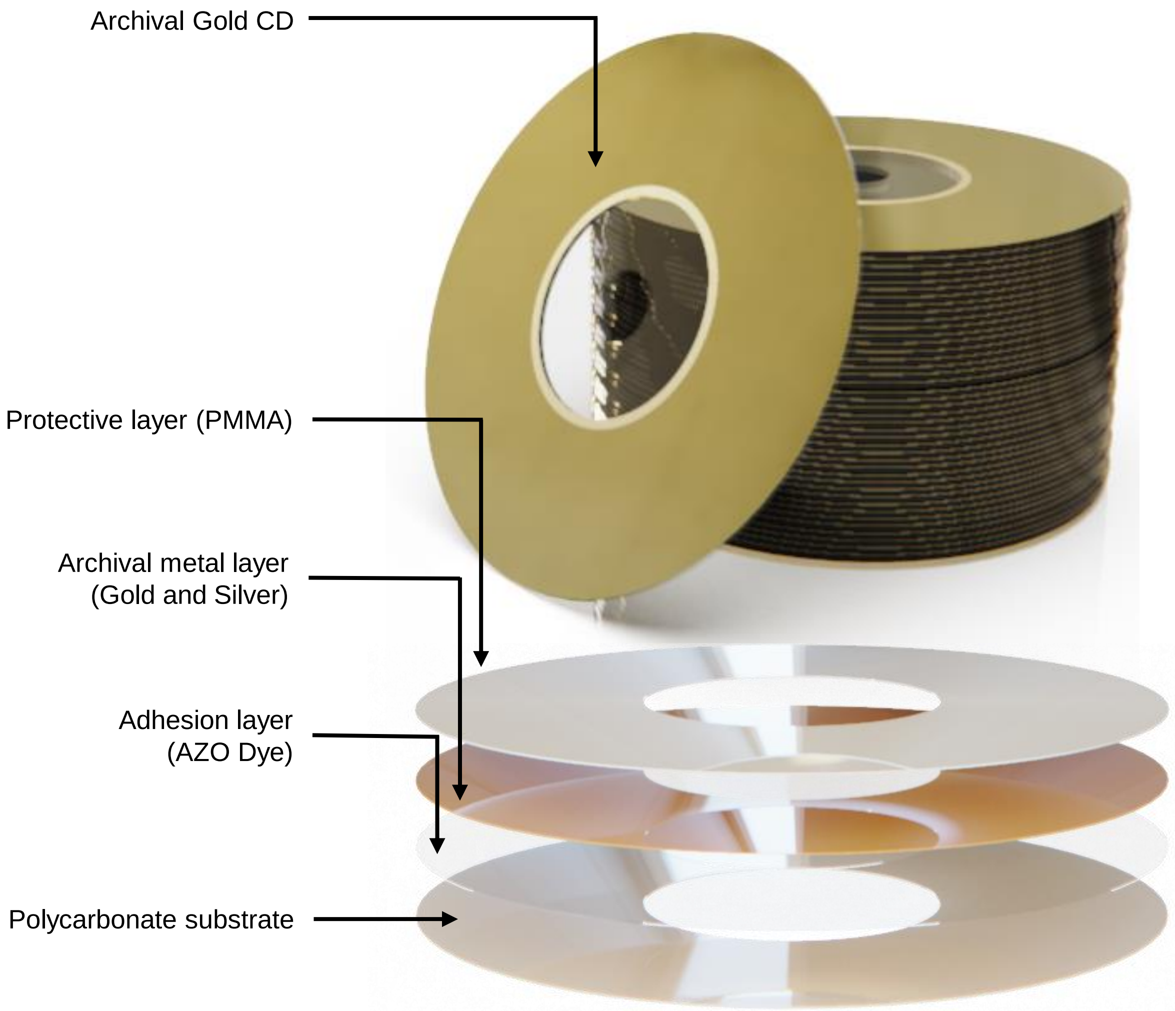

Supplementary Fig. 5. Structural schematic of an archival gold CD.

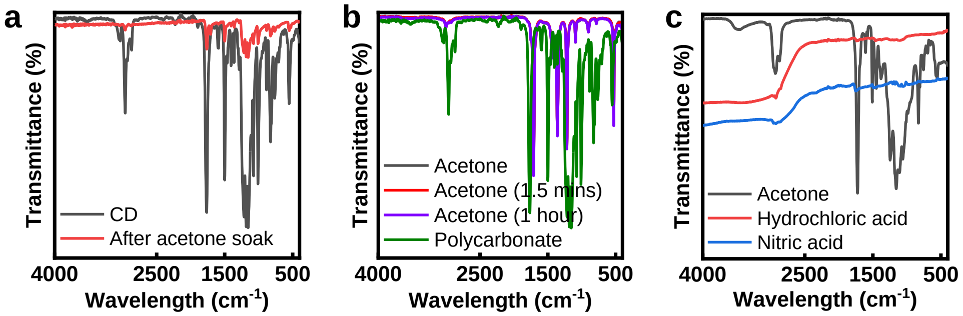

Supplementary Fig. 6. (**a**) FTIR spectrum from the substrate polycarbonate layer, before and after soaking the CD in acetone. (**b**) FTIR spectrum of acetone solution after soaking the CD. Acetone vs. acetone (after 1.5 minutes of the CD soaked) vs. acetone (after 1 hour of the CD soaked) vs. polycarbonate. (**c**) FTIR spectrum of the protective layer after soaking in acetone, hydrochloric acid, and nitric acid.

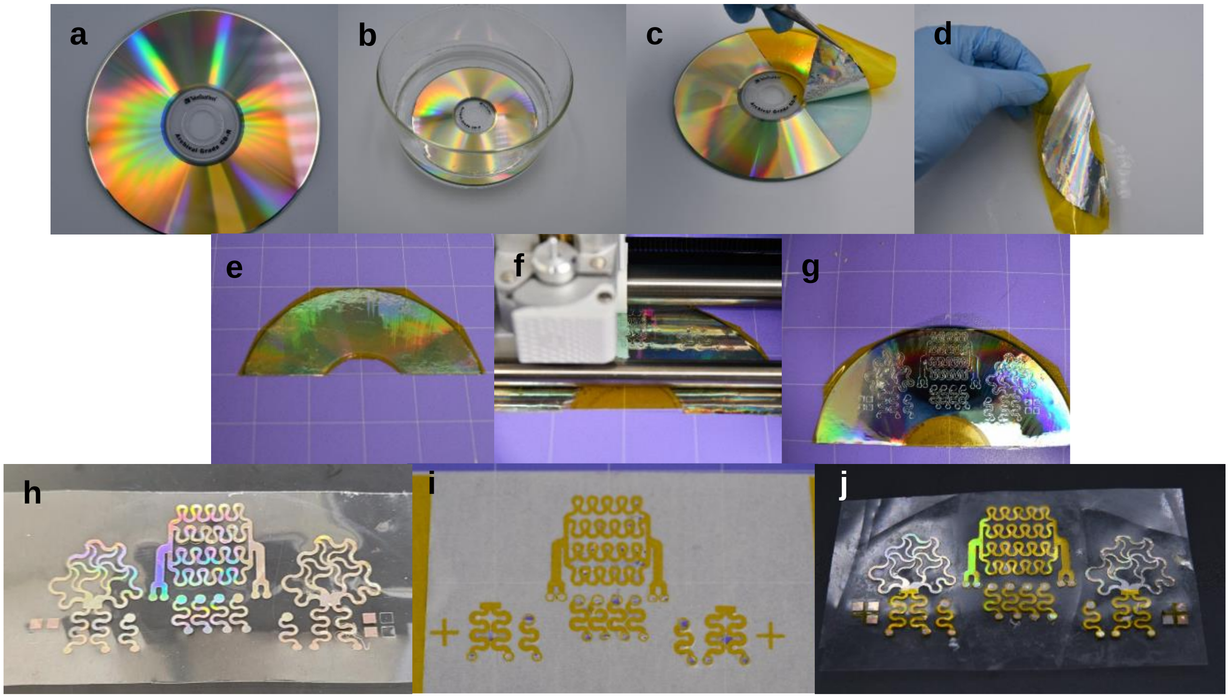

Supplementary Fig. 7. The fabrication process of the UCDEs. (**a**) Archival gold CD. (**b**) Soaked in acetone. (**c**) Harvesting the metal layer from the CD with PI tape. (**d**) Image of the harvested metal layer. (**e**) The PI side adhered to tattoo paper and then placed on the cutting mat. (**f**) Patterning with the cutting machine. (**g**) Post patterning. (**h**) Removal of excess. (**i**) Image of the insulation layer post cutting and after removal of the excess. A similar process is administered with the PI tape from steps **e**–**h**. The PI tape adhered onto water-soluble tape as a temporary substrate. (**j**) Full UCDE device. The PI tape is laminated over the patterned metal layer.

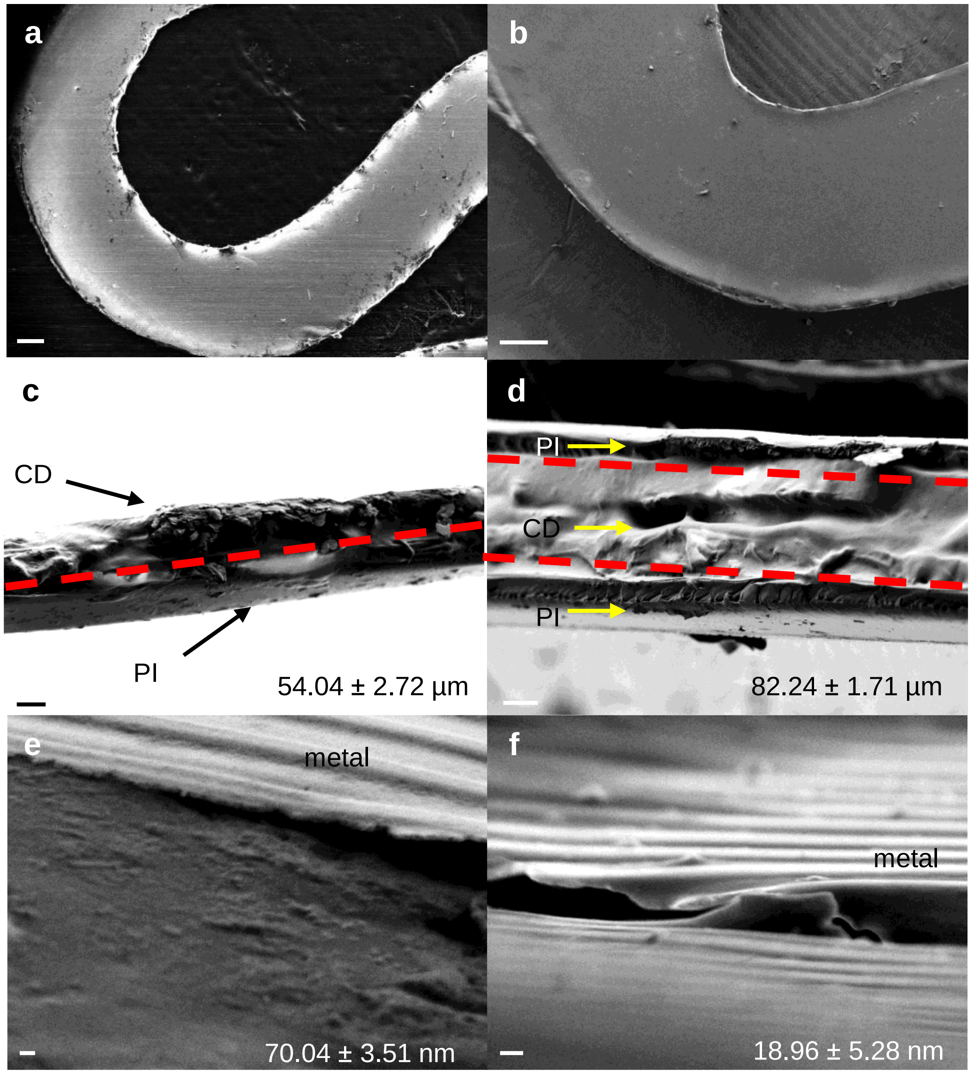

Supplementary Fig. 8. SEM images of UCDEs. (**a**) The metal layer, post patterning (scale bar, 200 µm). (**b**) Full encapsulation with PI (scale bar, 200 µm). (**c**) Cross-sectional image of the PI and CD metal (scale bar, 20 µm). (**d**) Cross-sectional image after full encapsulation with PI (scale bar, 20 µm). Metal layer remaining after (**e**) hydrochloric acid and (**f**) nitric acid soak (scale bar, 200 nm). The thickness of the metal layer is denoted in the bottom right of the image.

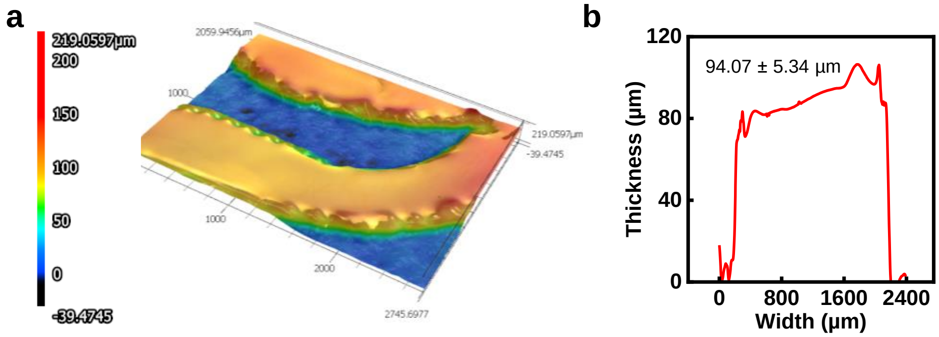

Supplementary Fig. 9. Optical profilometer analysis of UCDEs post full insulation. (**a**) Three-dimensional profile of UCDEs. (**b**) Thickness profile vs. width of UCDEs.

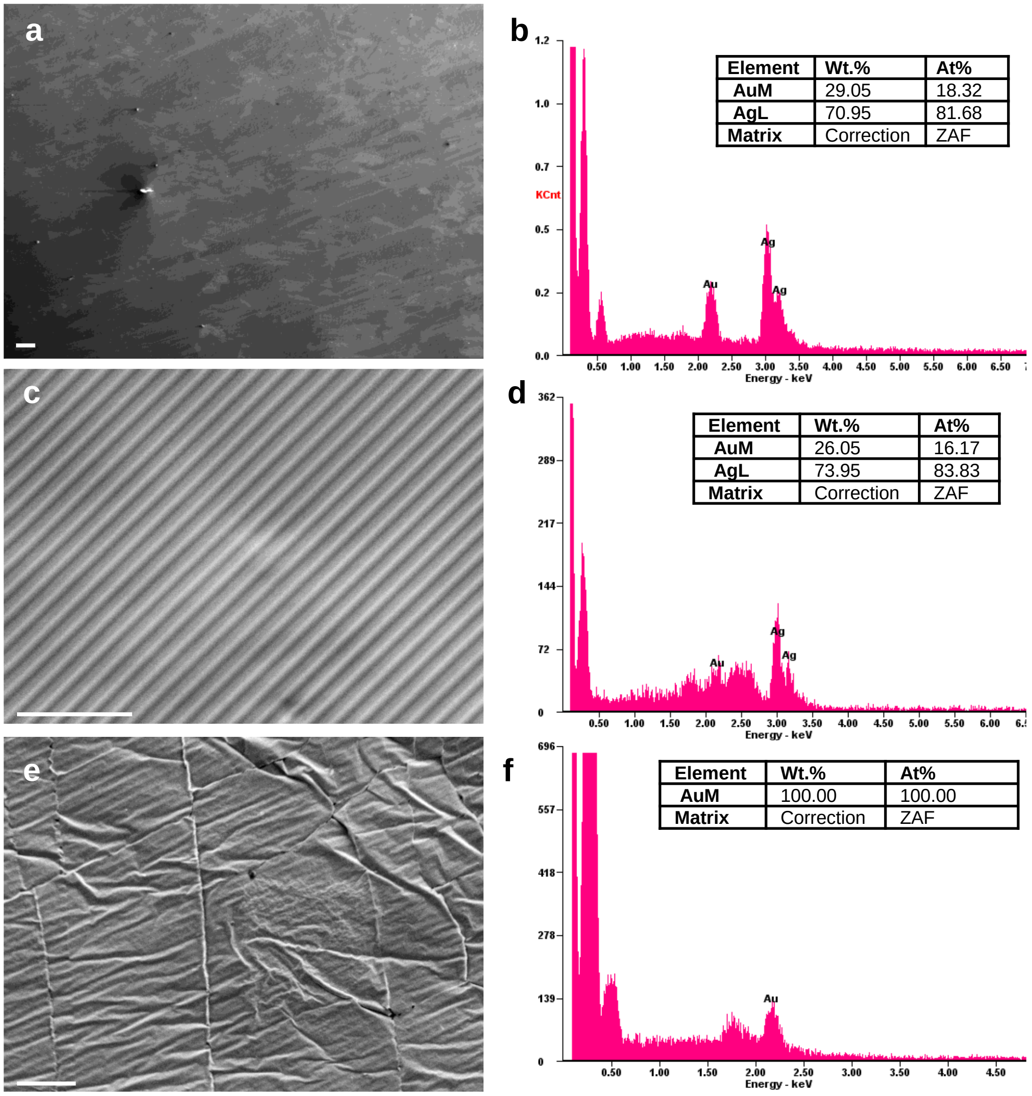

Supplementary Fig. 10. SEM images and EDS analysis of harvested metal films from CD. Acetone soak (**a**) SEM image (scale bar, 100 µm) and (**b**) EDS spectrum. Hydrochloric acid soak (**c**) SEM image (scale bar, 10 µm) and (**d**) E DS spectrum. Nitric acid soak (**e**) SEM image (scale bar, 10 µm) and (**f**) EDS spectrum.

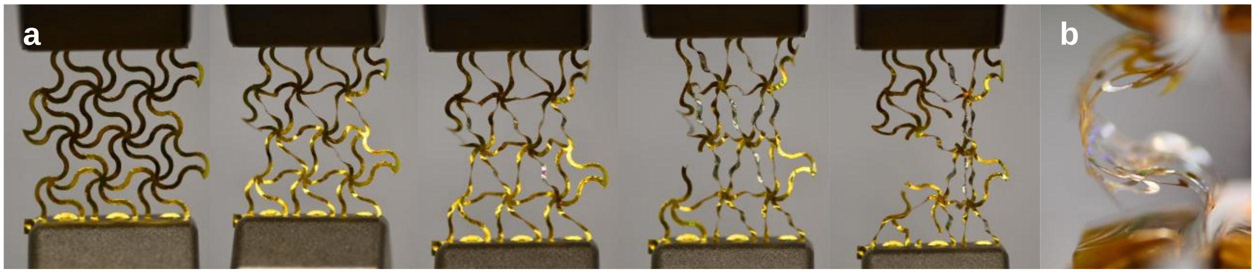

Supplementary Fig. 11. Mechanical response of patterned CD, UCDEs. (**a**) Images of tensile testing. (**b**) image of bending.

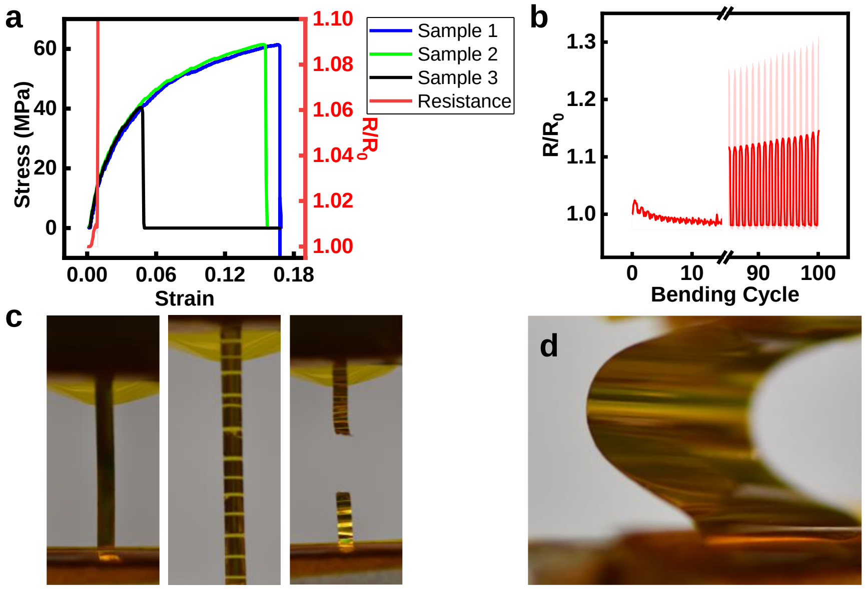

Supplementary Fig. 12. Mechanical properties of unpatterned CD electronics. (**a**) Stress and strain vs. electrical performance and (**b**) resistance vs. cyclic bending of unpatterned samples are presented as the average and standard error of means. Images of the unpatterned samples under (**c**) stretching and (**d**) bending.

Supplementary Table 1. Mechanical properties pattern and unpatterned samples.

Sample	Young's Modulus	Yield Strength	Ultimate Tensile Strength	Yield Strength Elongation (%)	Elongation (%)
**Patterned UCDEs**	5.59 ± 0.16 MPa	0.95 ± 0.07 MPa	0.99 ± 0.06 MPa	62.36 ± 1.81	129.24 ± 3.43
**Unpatterned CD Electronics**	1.88 ± 0.02 GPa	12.85 ± 0.91 MPa	54.47 ± 6.99 MPa	0.09 ± 0.05	12.39 ± 3.76
**PI Tape**	3.60 ± 0.60 GPa	32.45 ± 8.80 MPa	0.16 ± 0.02 GPa	0.88 ± 0.23	24.40 ± 6.52

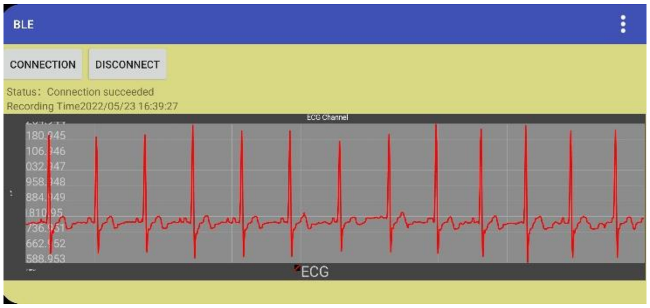

Supplementary Fig. 13. Screenshot of the smartphone application, recorded ECG signal.

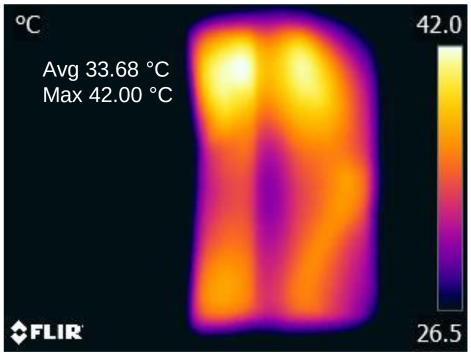

Supplementary Fig. 14. IR image of commercially available hand warmers (Hot Hands).

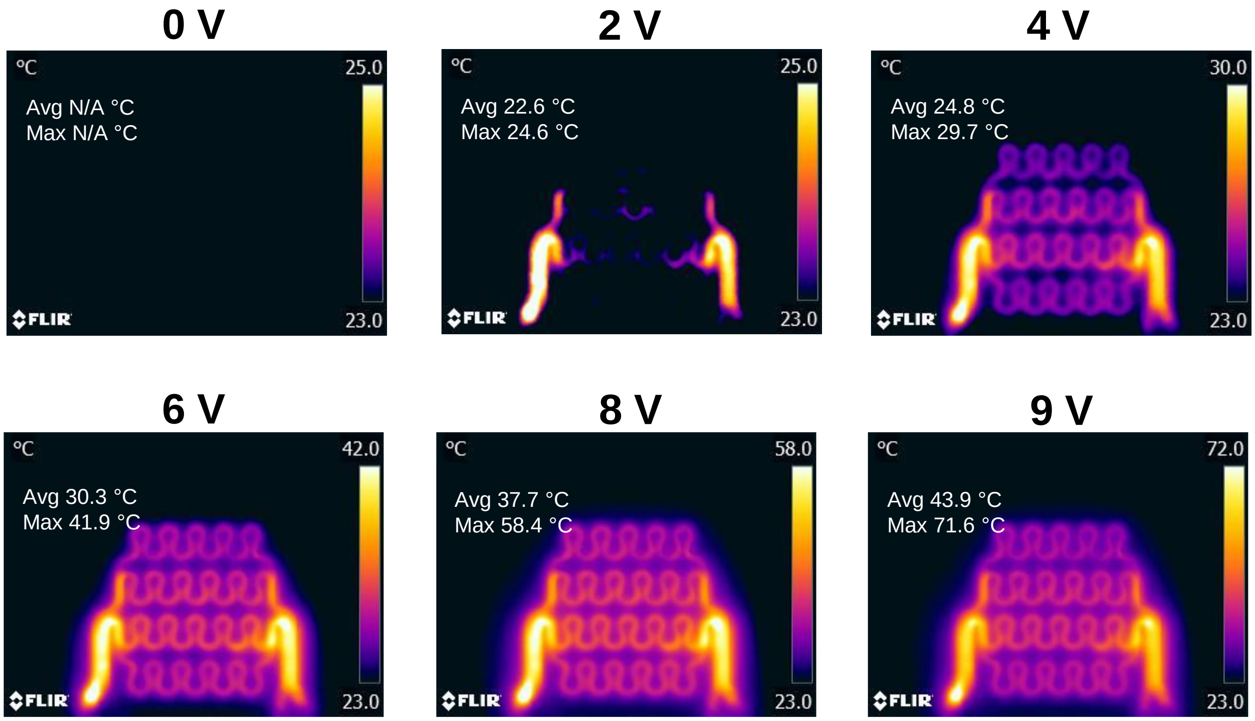

Supplementary Fig. 15. Larger, stretchable UCDE heater (3.5 cm width).

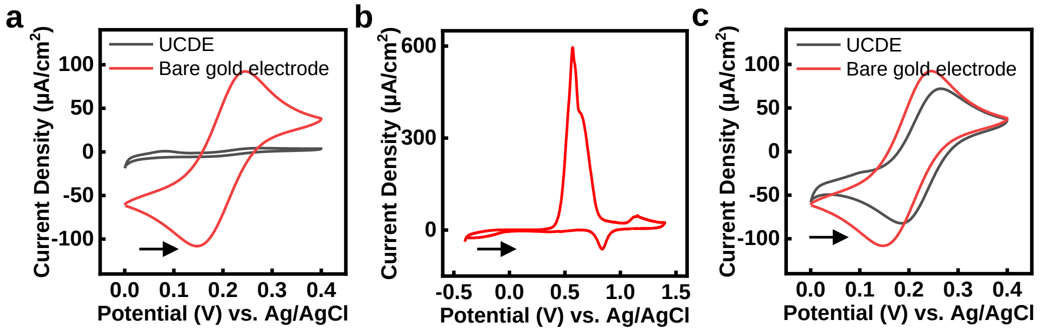

Supplementary Fig. 16. Characterization of the modified UCDE electrode before and after electrochemical cleaning. (**a**) Cyclic voltammetry at 50 mV/s of UCDEs (before cleaning) and bare gold electrode vs. Ag/AgCl (1 M KCl) in PBS (pH 7.4) with 5 mM $K_3Fe(CN)_6$. (**b**) Electrochemical cleaning of UCED vs. Ag/AgCl (1 M KCl) in 0.1 M H_2SO_4 at 25 mV/s. (**c**) cyclic voltammetry at 50 mV/s of UCDEs (after cleaning) and bare gold electrode vs. Ag/AgCl (1 M KCl) in PBS (pH 7.4) with 5 mM $K_3Fe(CN)_6$.

Supplementary Table 2. The characteristics of the fabricated UCDE Ag/AgCl reference electrode vs. a commercial Ag/AgCl (1 M KCl) reference electrode.

	1 mM $K_3Fe(CN)_6$ 1 M KCl			
	$E_{1/2}$(mV)	Δ(comm-fab) (mV)	ΔE (mV)	I_pa/I_pc
**Commercial**	261.00		74.00	1.54
**Fabricated**	246.50	14.50	83.00	1.17

	1 mM $K_3Fe(CN)_6$ 0.1 M KCl			
	$E_{(1/2)}$(mV)	Δ(comm-fab) (mV)	ΔE (mV)	I_pa/I_pc
**Commercial**	230.50		87.00	1.98
**Fabricated**	213.00	17.50	90.00	0.98

	1 mM $K_3Fe(CN)_6$ 0.01 M KCl			
	$E_{(1/2)}$(mV)	Δ(comm-fab) (mV)	ΔE (mV)	I_pa/I_pc
**Commercial**	217.00		106.00	1.13
**Fabricated**	193.45	23.55	101.10	0.70

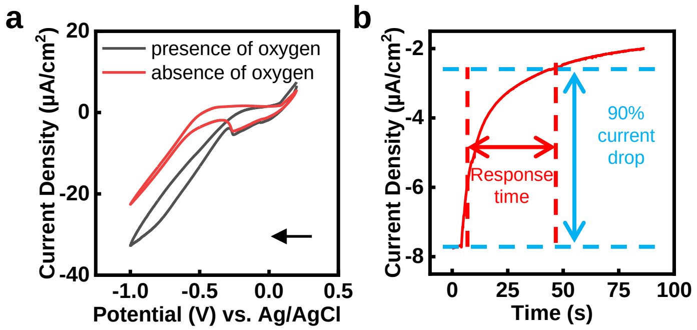

Supplementary Fig. 17. Characterization of the UCDE oxygen electrode. (**a**) Cyclic voltammetry of the UCDE oxygen electrode in the presence and absence of dissolved oxygen. (**b**) Sensor response time from oxygen saturated state to depleted state.

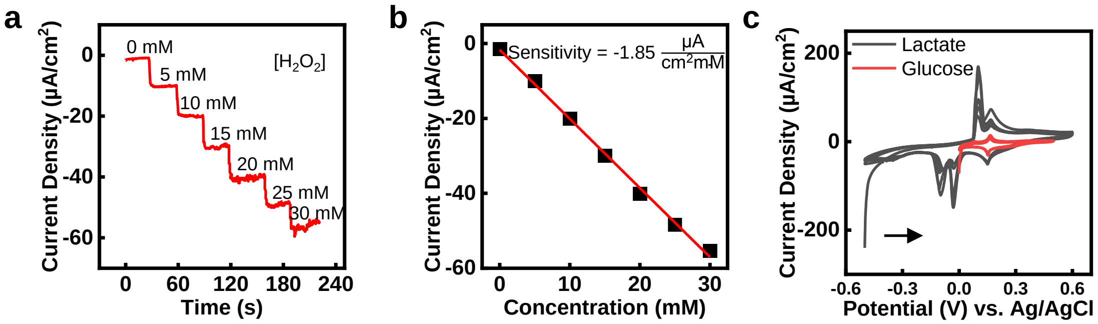

Supplementary Fig. 18. Characterization of the Prussian Blue modified UCDE electrode. (**a**) The amperometric i–t curves of the Prussian Blue, H_2O_2 detection sensor. (**b**) Calibration curve of the current density vs. H_2O_2 concentration. (**c**) Electrochemical deposition of Prussian Blue via cyclic voltammetry vs. Ag/AgCl (1M KCl).

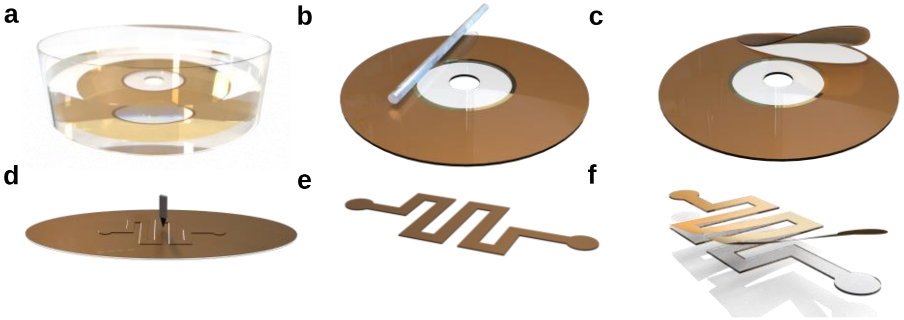

Supplementary Fig. 19. Upcycling CDs into Transient, Biodegradable Electronics. (**a**) Soaked in nitric acid. (**b**). Bar coated with a biodegradable polymer and cured. (**c**) Peeled off biodegradable polymer and gold layer from the CD. (**d**) Patterned with a mechanical cutter. (**e**) Removed excess and yielded a biodegradable device. (**f**) The two-layer layout of the biodegradable device.

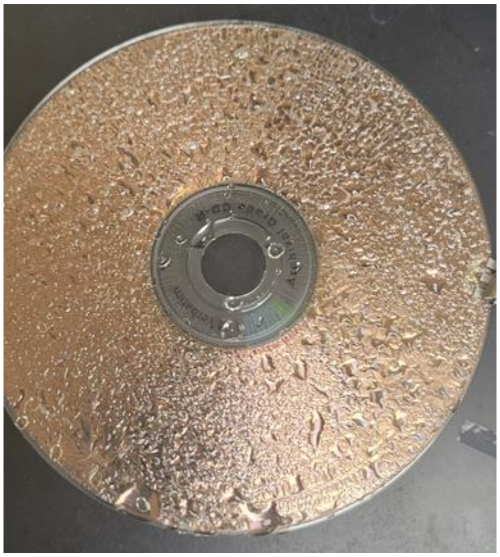

Supplementary Fig. 20. CD after the nitric acid soak.

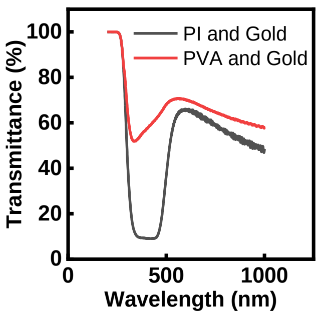

Supplementary Fig. 21. UV-Vis of the biodegradable PVA-based UCDE device.

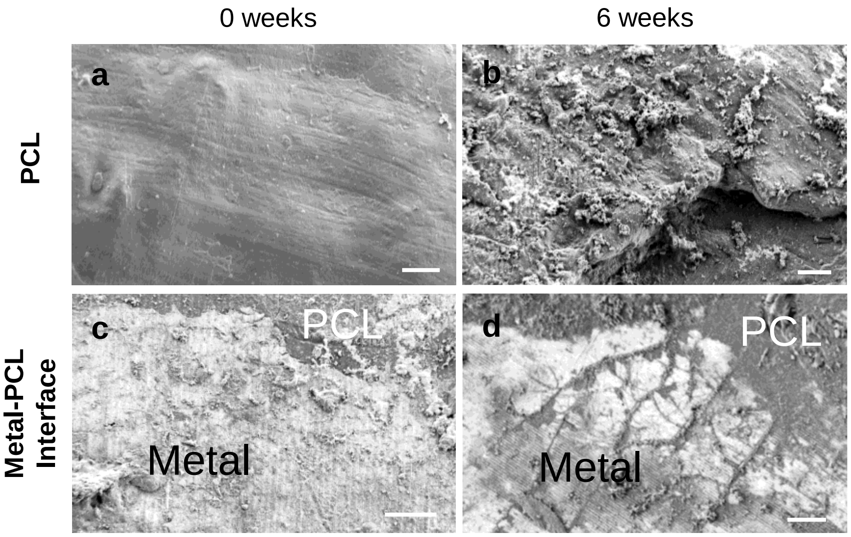

Supplementary Fig. 22. SEM images of PCL resistor degradation in PBS (7.4 pH) at 37 °C. PCL substrate at (**a**) 0 weeks and (**b**) 6 weeks. Metal-PCL interface at (**c**) 0 weeks and (**d**) 6 weeks. Scale bar 10 μm.

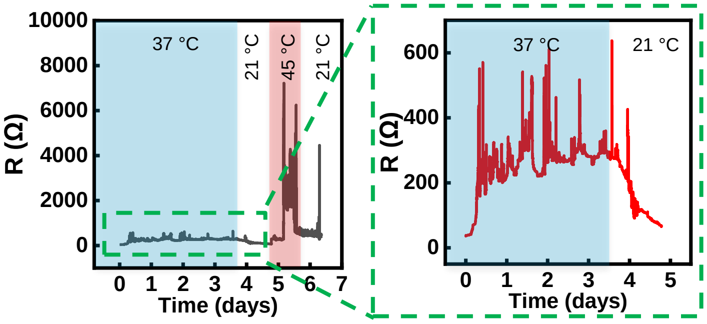

Supplementary Fig. 23. Long-term resistance stability of transient UCDE on PCL matrix. Soaked in PBS (pH 7.4) at 37 °C and 45 °C.

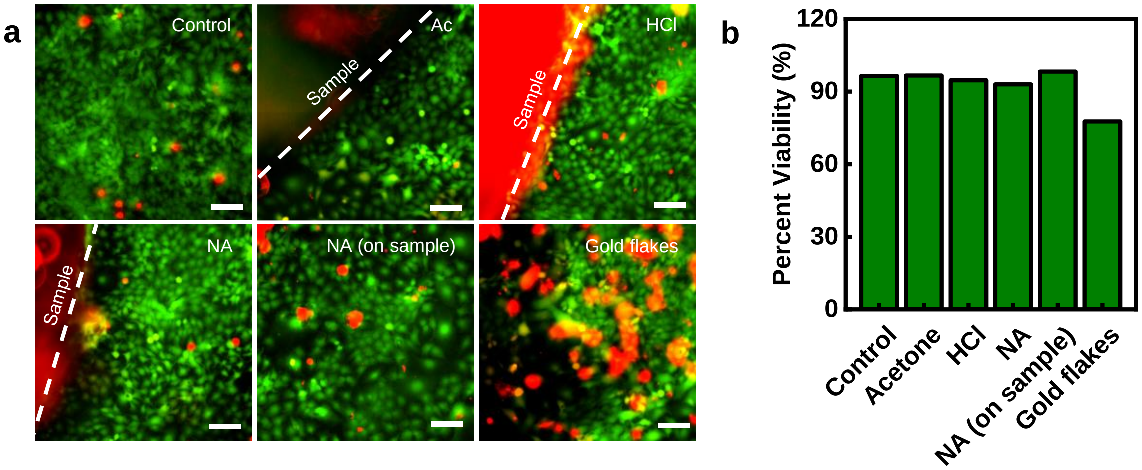

Supplementary Fig. 24. Biocompatibility of UCDEs. (**a**) Fluorescent imaging of live/dead stained HaCaT cell cultured for 7 days (scale bar, 100 µm). Sample group of the soaking method for UCDEs: Acetone (Ac), Hydrochloric acid (HCl), Nitric acid (NA), Na (image directly on the sample), and gold flakes. (**b**) cell viability at day 7. Analysis produced by fluorescent imaging viability counting.

References

1. Kang, S. K., *et al.* Bioresorbable silicon electronic sensors for the brain. *Nature* **530**, 71-76 (2016).
2. Armani, D. K. & Liu, C. Microfabrication technology for polycaprolactone, a biodegradable polymer. *Journal of Micromechanics and Microengineering* **10**, 80-84 (2000).
3. Drueckhammer, D. G. Acetone as a Recyclable Solvent. In: *Acetone: Biochemistry, Production and Uses* (ed Irenka Kozlowska, K. W.) 187-199 (Nova Science Publishers, 2018).
4. Zweckmair, T., *et al.* Recycling of Analytical Grade Solvents on a Lab Scale with a Purpose-Built Temperature-Controlled Distillation Unit. *Organic Process Research & Development* **21**, 578-584 (2017).
5. Jones, G. O., *et al.* Computational and experimental investigations of one-step conversion of poly(carbonate)s into value-added poly(aryl ether sulfone)s. **113**, 7722-7726 (2016).
